# Cancer in Africa: the way forward

**DOI:** 10.3332/ecancer.2019.953

**Published:** 2019-07-25

**Authors:** Peter Boyle, Twalib Ngoma, Richard Sullivan, Otis Brawley

**Affiliations:** 1 International Prevention Research Institute, 95 cours Lafayette, 69006 Lyon, France; 2 Strathclyde Institute of Global Public Health, Le Campus, Bâtiment l'Australien, 18 chemin des Cuers, 69570 Dardilly, France; 3 Muhimbili University, Mbezi Beach Block J Plot 59, PO Box 31658, Dar Es Salaam, United Republic of Tanzania; 4 Kings Health Partners Comprehensive Cancer Centre, School of Cancer Sciences, Institute of Cancer Policy, King's College London, London SE1 9RT, UK; 5 Johns Hopkins University, 3801 Canterbury Road, Baltimore, MD 21218, USA

**Keywords:** oncology, Africa, policy, disparities

## Abstract

While progress in oncology has been remarkable in recent decades, not every cancer patient is benefitting from the advances made in treating their disease. The contrast in diagnosis, treatment and its outcome between high-resource and low-resource countries is dramatic. Africa presents an enormous challenge with population growth and life expectancy increasing in many countries as the toll of AIDS and other communicable diseases declines. However, there has been little investment in capacity of any sort to deal with the current cancer problem, never mind the rapid increase in incidence which is underway. This is a critical area for investment and not only of a purely financial nature. It is bad to have cancer and worse to have cancer if you are poor. The gap between rich and poor, highly educated and less educated and the North–South divide is substantial and continuing to grow. Radical solutions are urgently needed: the status quo is not an appropriate response to the current situation. Recognising that no single government or source of philanthropy has the means to solve this problem, new models are needed to cope with and improve this situation.

## Introduction

The articles in this special issue provide some synthesis of the situation of cancer in Africa from individuals working on the ground. These are their observations about their work, their working conditions and their hopes for their patients and for the future of oncology care. It provides a companion volume to ‘The State of Oncology in Africa, 2015’ Report [1].

The current situation of cancer in Africa is outlined in this volume in the words of cancer specialists working in Africa and international experts who work closely with Africa and African colleagues. Overall, it paints a shocking, deplorable situation. However, hope for a better future comes from some high-quality, sustainable projects, such as in Eldoret (AMPATH Oncology) [2], the work of Hospice Africa Uganda, the collaborations between Crumlin Children’s Hospital (Dublin, Ireland) and Muhimbili University of Health and Allied Sciences (Dar-es-Salaam) Tanzania, the development by UNC-Zambian colleagues of scalable surgical services for women with cancer [3] and the Breast Health Global Initiative (BHGI). These, and others, contribute in a positive manner towards cancer control on the Continent. Another major source of hope is the remarkable dedication of those doctors, nurses and ancillary staff who work in Africa in conditions which would not be tolerated in high-income countries. Progress against cancer needs to be made through and with general health systems strengthening, the attainment of Universal Health Coverage and delivery of the sustainable development goals. There is a lack of cancer treatment centres [4]. These are huge challenges against a backdrop of significant social, economic and political fragility. And as we have tragically seen in the cases of Sierra Leone, Guinea and Liberia, the battle against the ravages of infectious diseases (Ebola) continues.

## The challenge: what could be done?

Despite the absence of accurate, population-based data from the majority of countries, all estimates indicate that the global cancer burden has doubled over the last 25 years and is set to double again before 2030 [5]. Not only have the incidence and the mortality increased, but with more and more patients alive within 5 years of diagnosis, the prevalence has been growing at an even quicker rate. This is of substantial economic importance, since five years after diagnosis is the time when many cancer patients continue to receive active treatment and intense follow-up.

Simultaneously, these recent decades have witnessed a remarkable improvement in many aspects of oncology from better understanding the causes, both lifestyle and biological, and enormous progress in developing more effective treatments for many forms of cancer. Progress has been made in each of the four Pillars of Oncology (Table 1) although, tragically, disparities exist at many levels of society and not every cancer patient has access to these modern advances. Even fewer of these advances have been implemented for the benefit of patients across the African continent.

What requires to be done in Africa bears striking similarities to that needed elsewhere, but needs to be modified to cope with further challenges presented by the unique situations across Africa. This is a continent of extraordinary diversity and richness, culturally, linguistically (there are around 2,510 languages spoken across the continent) and from socio-economic perspectives.

Prevention and Early detection are of major importance increasingly for the future: such programmes should be initiated now. In Africa, the critical need at present is for treatment and palliation.

### Treat all Cancers that can be treated

At the present time, scientific knowledge about cancer has never been greater although there remain many aspects of the disease which are not yet fully understood, with treatment resistance developing under primarily effective treatment constituting one of the major challenges and resulting in the incurability of mainly advanced disease. Despite great progress in developing cancer treatments in the past decades, many cancers are difficult to manage and frequently still carry a poor prognosis.

In high-resource countries, although two out of three people live at least 5 years after a cancer diagnosis, one cancer patient in three does not survive this long. Cancer, even at a particular anatomic site, is not one disease but an entity of highly complex individual diseases with various molecular and genetic characteristics and the resultant various prognoses which has led to the definition or at least exploration of various treatment modalities. The cancer problem is far from being solved although as a consequence from recent diagnostic and therapeutic advances, significant progress has been made in high-resource regions. In lower-resource settings, cancer survival rates are much poorer although demonstrating wide variations [6].

As an example, the evolution of treatment for breast, colorectal and prostate cancer, has considerably extended the armoury of treatments available for physicians and patients with these diseases. It is a similar pattern for many other forms of cancer. With each new therapy demonstrated to have efficacy in treating malignant disease, the overall survival time of the group of patients with the disease in question, improves. Alas, this only applies to the countries at the highest resource level since each new treatment is expensive and frequently requires specialist facilities to identify suitable patients for the treatments and also to deliver and monitor the treatment delivery.

Despite great improvements in the treatment of cancer in the past 30 years, many low-income countries have yet to see and/or benefit from the advances in surgery, radiotherapy and chemotherapy [7]. Since most patients in low-income countries present with advanced stage disease, conservative surgery which is now the norm for many cancers (such as breast) in high-income countries is out of question in low-income countries and the most that can be done is frequently very basic. Furthermore, in most low-income countries, the lack of surgeons, lack of hospitals equipped with operating theatres and patients’ inability to access and pay for surgical services pose real problems. Large advanced, inoperable cancers for which the only realistic option is palliative care are still a common occurrence in most low-income countries.

In most low-income countries, radiotherapy is either not available or in short supply. Exemplifying the stark difference between poor and rich nations regarding radiotherapy facilities is Austria, which houses the International Atomic Energy Agency headquarters. Austria possesses ONE radiotherapy machine for every 200,000 people or fewer, while many low-resource countries like Tanzania have only ONE radiotherapy machine for up to ten million people or more. Some of the world’s poorest nations have no radiotherapy facilities whatsoever [8] and those that have a facility frequently find that the machine is broken.

Abdel-Wahab *et al*. [9] made a longitudinal assessment of the status of radiation oncology resources in Africa to measure the extent of the problem and the effects of programmes designed to enhance radiation services on the continent. Radiation oncology departments in Africa were surveyed through the Directory of Radiotherapy Centres, and this information was supplemented by that available from International Atomic Energy Agency Regional African and Interregional project reports for 2010. Of 52 African countries included, only 23 are known to have teletherapy. These facilities are concentrated in the southern and northern states of the continent. Brachytherapy resources (high-dose rate or low-dose rate) were only available in 20 of the 52 African countries. Although progress has been made in the establishment of radiation oncology services in some countries, a large need still exists for basic radiation services, and much resource mobilisation is needed for services to keep pace with the burgeoning populations of many countries.

Furthermore, even in those low-income countries which have radiotherapy treatment machines, these machines are mainly cobalt 60 machines which are out of working order for a significant portion of time. These countries have no linear accelerators which have electrons option or Multi Leaf Collimators although much can be achieved using a cobalt machine [10]. The general experience in low-income countries is that most cancer patients would require radiotherapy to the primary and/or metastatic sites but having cobalt 60 machines only, makes it impossible to implement radiotherapy protocols to the standards available in high-income countries. Lack of radiotherapy facilities in low-income countries is a challenge which needs to be solved in order to improve the care of cancer patients [11].

The important role of chemotherapy in the treatment of many forms of cancer is undisputed. However, the chemotherapy drugs used in treating cancer which are normally widely available in high-income countries are in most cases not available in low-income countries. These drugs are frequently very expensive, unaffordable to the majority of patients in low-income countries and difficult to administer in low-resource settings. Prescribing and delivering chemotherapy is frequently complex and is not as simple as writing a prescription for drugs, such as anti-hypertensives or statins, and letting the patient then medicate at home. In addition, there needs to be continual monitoring and assessment of patients receiving chemotherapy.

The delivery of cancer chemotherapy in Africa is further hindered by widespread lack of healthcare professionals skilled in administering chemotherapeutic agents, access to laboratories for blood count analyses and effective antiemetic treatments. It is also important to note that most chemotherapeutic regimens have been field tested in clinical trials in high-income countries and that there is a possibility that treatment results from high-income countries are not generalisable to low-income countries, where the infrastructure, supportive care, patient and tumour characteristics differ markedly [12]. In a situation like this, local clinical trials to establish what works best in low-income countries are highly recommended rather than embracing the one size fits all notion and the dangerous trend that more expensive and newer drugs are better.

Progress in cancer treatment outcome has taken a step forward with the introduction of personalised medicine and the consequent development of targeted therapy. For this to be successful, cancer patients need to present to their physician at a stage when the disease is not at such an advanced stage that cure and treatment are not possible. In many instances, this will rely on the widespread availability of adequate diagnostic facilities. There needs to be knowledge not simply of the histology of the tumour but knowledge of the presence of the requisite biomarker. This requires knowledge of biomarkers in the population derived from the presence of high-quality biobanks and the availability of the technology for measuring biomarkers relevant for stratified medicine.

There must also be adequate treatment facilities available, including surgery, radiotherapy and the medical oncology and palliative care skills for delivering the chemotherapy. Obviously, the drug needs to be available and affordable. The manpower to support the entire system needs to be trained and in place and there needs to be adequate funding to support these new developments.

Critical requirements are the availability of biobanks and laboratories in which the biomarkers necessary for identifying the most appropriate therapy can be reliably measured. There is an acute absence of biobanks in lower-income countries and the routine collection of biological samples and their storage in adequate facilities is not a common feature [13]. The absence of Biobanks means that little is known about the presence or frequency of biomarkers in lower-resource countries. In the absence of Biobanks, there is a lack of the technology for measuring biomarkers. This includes lack of the necessary equipment, biological reagents and laboratory skills to measure biomarkers and to exert quality control standards.

Another aspect of treatment that is needed but poorly provided in Africa, and other low-income countries, is palliative care. There is a lack of health care professionals skilled in palliative care. Even for those countries with health workers with proper training in palliative care, the palliative care workers in these countries are restricted in their ability to provide comfort care and pain relief for cancer patients, especially as part of end-of-life care because many common and effective pain medications, such as morphine, are not readily available [14].

The fact that in low-income countries access to health care is a major problem, cancer awareness is limited, cultural barriers are plenty, patients present with advanced disease and there is lack of the fundamental infrastructure that is required to be able to copy what is being done in developed countries compels low-income countries to develop alternative strategies in the treatment of cancer.

Since the development and implementation of these strategies extends beyond the capacity of most low-income countries, sustainable outside assistance is required. An opportunity has been created by the BHGI. Among other breast cancer developments [15], the BHGI, co-sponsored by Fred Hutchinson Cancer Research Centre and Susan G. Komen for the Cure has developed study evidence-based, economically feasible and culturally appropriate Guidelines for breast cancer for low- and middle-income countries to improve breast health outcomes and access to breast cancer screening, detection and treatment. These guidelines which were developed making sure that they are not defining a 'lowered' standard of care for that country are readily available and user friendly [16, 17]. Low-income countries should, therefore, put in place proper strategies to implement the guidelines to the greatest extent possible.

It is estimated that the annual number of new cases of cancer in Africa will grow to more than one million in the next 5 years. Together with the immense loss in human life, there is a considerable economic setback attached to this number. However, most African nations are far from adequately scaling up their capacity to control cancer. Stefan [4] reviewed the published data on the existing cancer control resources in Africa: the first combined effort looking at all the resources available on the continent regarding cancer care. The total number of 102 cancer treatment centres, including general oncology centres, gynaecologic oncology or other single-organ malignancy units, and paediatric oncology and palliative care establishments, is not sufficient to cover the increasing needs of the African population affected by cancer [4].

Today, the great majority of cancers have some positive degree of response to appropriate treatment. When viewed in a historical context, recent progress has been remarkable in developing effective therapies which frequently prolong life and substantially improve the quality of the remaining lifespan. This comes from a variety of factors including the rapid increase in knowledge of what constitutes malignant disease and the biological processes which cancer involves. Cancer treatments have improved substantially. Surgery is less mutilating; Radiotherapy is more effective; Chemotherapy is also more effective and Nutrition of patients is improving. However, many patients around the world do not have access to these modern therapy regimes for a variety of reasons which encourage the growth of this disparity.

One way of dealing with the current disparities in treatment of cancer is to view cancer treatment and cancer control in general as a human rights issue. In so doing, all the world leaders and organisations have to work together to defend the human right of access to cancer care just like the way they defend democracy. Low-income countries have to set their own national cancer control agenda with the high-income countries and international organisations in a supporting role—not the other way around. Every cancer patient, no matter their situation, has a right to the most appropriate treatment for their condition at the right time.

### Provide palliation whenever palliation is needed

There have been major improvements in all the aspects of Palliative, Supportive and Terminal Care in the past decades although it has been surprisingly slow to be rolled out in every high-resource country. It is also disquieting that the little that is known about the quality-of-life of cancer patients comes from a remarkably small number of randomised trials. Palliation is needed not only for pain control in the final moments of life, but should be available at every part of the cancer pathway: at the time of surgery, radiotherapy and during chemotherapy. In Africa, the situation is frankly appalling and there are very few trained in palliative care. There are around 30 countries without a single radiotherapy machine (which is useful for pain control) and the same number of countries where the importation of opioid medication is forbidden. Where opioids are available, the dose used can vary by several hundred-fold. Paracetamol is not an effective medication for the control of severe cancer pain, but that is all that is available in too many countries.

Patients living with and dying from cancer have the fundamental right to do so with dignity and comfort irrespective of their disease or where they live. The contrast between high-income and low-income countries in terms of supportive, palliative care and terminal care is even greater than for cancer treatment services. This year, more than 8,000,000 people internationally will die as a direct result of cancer, many of whom will have had their lives substantially shortened, rising to 17 million people by 2030 [5]. The predictable effects of advancing cancer challenge health systems to plan for and resource the relief of the suffering experienced by people and their caregivers as the disease progresses within a public health framework.

The continuing improvements in cancer prevention, early detection and treatment are still overshadowed by premature mortality as a result of cancer. In resource-rich countries, two out of every five people diagnosed with cancer will die prematurely. This can rise to nine out of ten people in resource-poor countries where late presentations and limited resources deliver poor survival rates and, frequently, deaths in atrocious circumstances.

The control of pain and suffering is central to health, and the right to health is stipulated in several International declarations. The Korean Declaration [18] states that ʻEvery individual has the right to pain reliefʼ The Cape Town Declaration [18] stated that the control of pain and symptoms is a human right, and, therefore, appropriate drugs should be available in every country in sub-Saharan Africa as a part of the essential drug list, including opioids, such as morphine. These drugs should be available and accessible. Sadly, this is not the case in Africa.

There are wide variations in the availability and quality of supportive and palliative care around the world and within countries. For many countries, the availability of opioids is limited or absent. In the period 2000–2002, 29 countries in Africa reported NO opioid availability and for the 18 countries reporting use, there was a 100-fold difference in the average defined daily dose of morphine. Another key element for palliative cancer care, particularly pain relief, is radiotherapy. Given that there are more than 30 countries without opioid medications and radiotherapy, hopes of a pain-free, dignified death for the majority of cancer patients is a priority that needs to be urgently addressed.

## The practical: what must be done

While progress in oncology has been remarkable in recent decades, and the future looks very encouraging, not every cancer patient is benefitting from the advances made in treating their disease. This is true even in high-resource countries where there are substantial differences in outcome according to the individual’s deprivation status. The contrast in diagnosis, treatment and its outcome between high-resource and low-resource countries is dramatic.

This is a particularly important issue since the pattern of cancer globally in the foreseeable future will be heavily dependent on what happens in China, India and Africa, where one half of the world’s population currently live and the populations of each are ageing quickly and have developed lifestyle habits conducive to increasing cancer risk. India has a long tradition in Cancer Care but faces the challenge of extending that care to their growing population. China faces similar challenges to India and has been making solid investment in training and infrastructure to cope with the huge problem the country is facing. Life expectancy has increased from 55 years ago to reach 75 at the present time and with it has come a rapidly increasing burden of cancer and other chronic diseases linked to ageing. Africa presents the biggest challenge with population growth and life expectancy increasing in many countries as the toll of AIDS declines. However, there has been little investment in capacity of any sort to deal with the current cancer problem never mind the rapid increase in incidence which is underway. This is a critical area for investment and not only of a purely financial nature.

It is bad to have cancer and worse to have cancer if you are poor. The gap between rich and poor, highly educated and less educated and the North-South divide is substantial and continuing to grow. Radical solutions are urgently needed: the status quo is not an appropriate response to the current situation. Recognising that no single Government or source of philanthropy has the means to solve this problem, new models are needed to cope with and improve this situation. It is impossible to avoid the conclusion that there is a need for a major significant increase in domestic expenditure on public health, a wider application of health structural funds from overseas development aid (ODA) to include non-communicable diseases (NCDs) and creative approaches to private–public partnership, involving a number of sources from different areas, to make the necessary progress with the briefest delay. The partnership needs the commitment of the pharmaceutical industry and the wide span of industries involved in the technology for diagnosis and treatment. It needs the commitment of governments and non-governmental organisations to be effective. Effective will be measured against the right of every patient with cancer to have the most appropriate treatment and care for their disease.

Working to improve health must cease to be viewed as a competition. Public and private organisations have an underlying suspicion of each other that must be overcome in the interests of improving cancer care and outcome worldwide. The situation as portrayed in this Report is dramatic and urgent and it behoves all parties to put this frequently deep-rooted suspicion behind them and develop an effective collaboration to improve this key aspect of public health throughout the world.

The current situation regarding Cancer in Africa is quite deplorable. Many patients do not seek medical advice. Those who do, do so when the cancer is at an advanced stage when cure is no longer possible. There is a lack of oncologists of all kinds, nurses and the necessary health professionals and technicians to support their work. There is a lack of treatment centres. There is a lack of treatments. Most countries do not have any Radiotherapy equipment. Most countries do not have access to opioid drugs for palliative care and pain control.

The situation is bound to get worse as the population grows and ages and cancer risk factors imported from high-resource countries begin to have their effect. The evidence is clear. Over the next decades, cancer will cause Africans to suffer and die in greater numbers; much greater numbers.

It is essential to move from a passive position to an active voice. We can turn our heads and walk away from this situation and betray all those wonderful clinicians, nurses and other personnel grasping with the overwhelming problem of cancer on the Continent. Or, we can do something.

Radical solutions are necessary: the status quo is not an option. There is hope in the sense that there are outstanding examples where effective and efficient oncological services function. There are clearly identified needs.

First of all, we need to train more oncologists and health professionals and provide the necessary infrastructure which is urgently needed to identify and treat patients. More general and specialist surgical capacity and manpower are critical. This includes concomitant enhancements in imaging and pathology.

Two, the drugs and equipment necessary to treat patients with cancer must be made available. We need to deliver, install and maintain adequate numbers of Radiotherapy machines in each country, including linear accelerators. It should be the right of cancer patients, no matter where they are to have access to the appropriate treatment of their disease.

Three, opioids must be available for controlling the pain of patients with terminal cancers (and other diseases). International Agencies, such as the International Narcotics Board, should make this a priority activity and come to agreements with Governments of countries where these are not available.

Four, since a third of cancers in Africa is currently caused by chronic infection, relevant vaccination programs must be funded and implemented continent-wide.

Five, information campaigns to wipe out stigma and misinformation must be conceived and disseminated.

Six, we must stop at nothing to improve access to healthcare and pathology services. The role of the Pharmaceutical Industry and others, such as device manufacturers, is critical.

Seven, we call upon high-quality cancer institutions, all over the world, to establish collaboration ventures with African cancer institutes and public health services.

Finally, we call upon international philanthropy to help fund these efforts.

## Conclusion

This is a call to African governments but also to the many foreign governments and international organisations who are distancing themselves from the crisis. Reports with idealised solutions are very well but that time has passed. The necessity now is for coordinated and sustainable action.

## Funding statement

This article was funded from internal sources within the International Prevention Research Institute.

## Conflicts of interest

The authors have no duality of interest to declare.

## Figures and Tables

**Table 1.** The four pillars of oncology.

I	Prevent all cancers that can be prevented
II	Treat all cancers that can be treated
III	Cure all cancers that can be cured
IV	Provide palliation whenever palliation is required
